# The Genomic Environment of *BRAF* Mutated and *BRAF*/*PIK3CA* Double Mutated Colorectal Cancers

**DOI:** 10.3390/jcm11175132

**Published:** 2022-08-31

**Authors:** Ioannis A. Voutsadakis

**Affiliations:** 1 Algoma District Cancer Program, Sault Area Hospital, Sault Ste. Marie, ON P6B 0A8, Canada; ivoutsadakis@yahoo.com or ivoutsadakis@nosm.ca; 2 Section of Internal Medicine, Division of Clinical Sciences, Northern Ontario School of Medicine, Sudbury, ON P6B 0A8, Canada

**Keywords:** genomics, signal transduction, molecular alterations, gastrointestinal cancers, kinase

## Abstract

Background: Colorectal cancer represents the most prevalent gastrointestinal malignancy. Prognosis of metastatic disease has improved in recent years with the introduction of effective systemic therapies, but mean survival remains in the range of two to three years. Targeted therapies based on specific molecular alterations in sub-sets of colorectal cancers have the potential of contributing to therapeutic progress. *BRAF* and *PIK3CA* are oncogenic kinases commonly mutated in colorectal cancers and can be targeted through small molecule kinase inhibitors. Methods: Clinical and genomic data from two extensive series of colorectal cancers were interrogated to define the molecular characteristics of cancers with *BRAF* mutations with and without concomitant mutations in *PIK3CA*. Results: Colorectal cancers that are *BRAF* and *PIK3CA* double mutants represent a small minority of about 5% of colorectal cancers in the two examined series of mostly localized disease. They also represent about one third of all *BRAF* mutated colorectal cancers. Most mutations in *BRAF* are classic V600E mutations. A high prevalence of MSI and CIMP is observed in *BRAF* mutated colorectal cancers with or without *PIK3CA* mutations. Mutations in tumor suppressors *FBXW7* and *ATM* display a higher prevalence in *BRAF* mutated cancers. The prognosis of *BRAF* mutated colorectal cancers with or without *PIK3CA* mutations is not significantly different than counterparts with wild type *BRAF*. This contrasts with the known adverse prognostic effect of *BRAF* in metastatic disease and relates to the different prevalence of MSI in mutant *BRAF* localized versus metastatic colorectal cancers. Conclusions: *BRAF* mutations are the defining molecular alterations in double mutant *BRAF* and *PIK3CA* colorectal cancers as determined by increased MSI and CIMP in *BRAF* subsets with and without *PIK3CA* mutations. Moreover, *BRAF* mutated cancers with and without *PIK3CA* mutations are characterized by the absence of *KRAS* mutations and a lower prevalence of APC mutations than *BRAF* wild type counterparts. Mismatch-repair-associated gene mutations display higher frequencies in *BRAF* mutated colorectal cancers. Despite the absence of prognosis implications of *BRAF* mutations in the studied cohorts of mostly localized cancers, such mutations could be prognostic in certain subsets. The presence of mutations in other genes, such as *ATM* and high MSI status present opportunities for combination therapies.

## 1. Introduction

*BRAF* is a serine threonine kinase which is part of the RAF/MEK/ERK cascade transducing signals from growth factors for cell proliferation and apoptosis inhibition [1]. Two other RAF homologues, ARAF and CRAF (also known as RAF1), exist in human cells encoded by distinct genes. *BRAF* is encoded by a gene at human chromosome 7q34 while genes encoding for ARAF and CRAF are located at chromosomes Xp11.3 and 3p25.2, respectively. The three proteins can homodimerize or heterodimerize with another protein of the family [2]. Wild type RAF proteins are activated by RAS to dimerize and phosphorylate kinases of the MEK family which in their turn activate ERK kinases. ERK kinases inhibit apoptosis and promote proliferation by activating transcription factors, the prototypic being the AP1 (Activating Protein 1) complex.

In contrast to other RAF homologues, *BRAF* is commonly mutated in cancers. The most common canonical mutations at position V600 are currently effectively targeted with small molecule inhibitors in malignant melanoma and other cancers [3]. Mutations in *BRAF* occur in about 10% of colorectal cancers and have been examined for therapeutic targeting with the same small molecule inhibitors used in melanoma [4]. A *BRAF* inhibitor is currently approved in combination with the anti-EGFR monoclonal antibody cetuximab for second line treatment of metastatic colorectal cancers with *BRAF* mutations [5]. However, as shown in the registration trial, only one in five patients respond to therapy, despite all patients bearing the targeted *BRAF* mutation [6]. Development of resistance leading to progression in responding patients is also the rule [7].

The other signal transduction cascade activated by growth factors and receptor tyrosine kinases is the PI3K/AKT/mTOR cascade. The cascade is triggered through the activation of kinase PI3K by diverse receptor tyrosine kinases, such as the Epidermal Growth Factor Receptor (EGFR) family receptors, the Fibroblast Growth Factor Receptor (FGFR) family receptors, the Platelet-derived Growth Factor Receptor (PGFR), and the Insulin-like Growth Factor Receptor (IGFR) [8]. Production of the lipid phosphatidylinositol-3,4,5-triphosphate in the cell membrane by activated PI3K enables proximity of kinase PDK1, the mTORC2 complex and kinase AKT, resulting in phosphorylation and activation of the latter [9]. AKT has several substrates involved in carcinogenesis and results in the promotion of cell proliferation and apoptosis inhibition. Through down-stream activation of the mTORC1 complex, AKT positively regulates protein translation and cell growth [10,11]. The most common mutation in the PI3K/AKT pathway is in the gene encoding for the alpha catalytic sub-unit of PI3K, *PIK3CA*, occurring in about one in four colorectal cancers [12]. Half of the activating mutations in *PIK3CA* occurring in colorectal cancers concern hotspots of amino acid positions E545, E542 and Q546 of the helical domain or position H1047 of the kinase domain, while the other half is distributed across the gene [12]. Mutations in *PIK3CA* are not mutually exclusive with *BRAF* mutations and occur in both *BRAF* mutated and wild type colorectal cancers. Thus, these mutations may play a role in resistance of a subset of *BRAF* mutated cancers to targeted therapies, through the extensive crosstalk of the KRAS/RAF/MEK/ERK and the PI3K/AKT/mTOR pathways [13,14]. For example, mTORC1 activation by PI3K/AKT provides a feedback inhibition of receptor tyrosine kinase signaling through Insulin Receptor Substrate proteins IRS1 and IRS2 and adaptor protein GRB10 (Growth Factor Receptor Bound protein 10) [15,16]. Other crosstalk points between the pathways are mediated by activation of mTORC1 and inhibition of TSC1 and TSC2 (Tuberous Sclerosis Complex 1 and 2) by MEK kinases and activation of PI3K by KRAS [17].

This paper examines colorectal cancers with *BRAF* mutations with and without concomitant *PIK3CA* mutations using data from published genomic studies. A detailed understanding of the molecular processes associated with these distinct subsets of colorectal cancers and additional molecular aberrations that support *BRAF* mutations in the neoplastic process is needed for improving therapeutic results.

## 2. Methods

The Cancer Genome Atlas (TCGA) colorectal cancer cohort study and the Dana Farber Cancer Institute (DFCI) cohort, two genomic studies with 594 and 619 included patients, respectively, were included in this analysis and analyzed separately due to differences in the genomic platforms used and different tools for molecular characteristics assignment [18,19]. TCGA includes data on both single nucleotide variants and copy number alterations, while the DFCI study provides only molecular information for single nucleotide variants. Both series used whole-exome sequencing for detection of somatic single nucleotide alterations. A third series including only metastatic colorectal cancers employed a targeted genomic panel and was used for exploration of the prevalence of *BRAF* mutation in primary versus metastatic site biopsies [20].

The current study used the cBioPortal for Cancer Genomics portal (cBioportal, http://www.cbioportal.org (accessed on 5 May 2022)), an open-source genomics site maintained by MSKCC and other academic institutions, for interrogation of the three studies of interest [21,22]. Subsets of cases with and without *BRAF* and *PIK3CA* mutations were examined in cBioportal for pertinent clinical and genomic characteristics. TCGA uses three different pipelines for single nucleotide variant calling, while the DFCI series used the MuTect algorithm [23]. TCGA provides two scores for the evaluation of global chromosomal instability of a sample, the Aneuploidy Score (AS) and the Fraction Genome Altered (FGA) score. AS was derived by summing the number of chromosome arms in each sample that have copy number alterations (gains or losses). A chromosome arm was considered copy number altered, gained, or lost, if there was a somatic copy number alteration in more than 80% of the length of the arm as calculated by the ABSOLUTE algorithm from Affymetrix 6.0 SNP arrays [24]. Chromosomal arms with somatic copy number alterations in 20–80% of the arm length are considered indeterminate (not called) and chromosomal arms with somatic copy number alterations in less than 20% of the arm length are considered not altered. The FGA was derived by source segment files and is calculated by summing the length of segments with log2 greater than 0.2 divided by the total length of all segments measured in the sample. The pathogenic implications of mutations in genes of interest were derived from the OncoKB knowledgebase [25].

Statistical comparisons of categorical and continuous data were carried out with the Fisher’s exact test or the χ^2^ test and the *t*-test or ANOVA (Analysis of Variance) test. Survival analysis was performed with construction of Kaplan–Meier survival curves from source data. The log rank test was used to compare Kaplan–Meier survival curves. All statistical comparisons were considered significant if *p* < 0.05.

## 3. Results

In total, 22 of 534 profiled colorectal cancer patients in the TCGA cohort (4.1%) had mutations in both *BRAF* and *PIK3CA* oncogenes. This represents 35% of the total number of cases (62 cases) with *BRAF* mutations. A total of 15 of the 22 patients had V600E *BRAF* mutations (with additional non-classical *BRAF* mutations in 2 of the 15 patients). Three patients had mutations at codons 594 and 597 of *BRAF* that are also considered pathogenic (categorized as classes II and III as opposed to codon V600 mutations that are categorized as class I) and 4 patients had other *BRAF* mutations of unknown significance. In 20 of the 22 patients, *PIK3CA* mutations that are considered pathogenic were present, while in 2 patients *PIK3CA* mutations were evaluated as of unknown significance. Three cases had *PIK3CA* mutations at hotspot amino acid position E545, 1 patient at position E542, and 10 patients had *PIK3CA* mutations at hotspot position H1047. The six remaining cases with *PIK3CA* pathogenic mutations were mutations in amino acid positions other than E545, E542, and H1047.

In the DFCI cohort, 35 of 619 patients (5.7%) had mutations in both *BRAF* and *PIK3CA* oncogenes. This represented 27.6% of the total number of cases with *BRAF* mutations in the DFCI cohort. In total, 28 of the 35 patients had classic V600E mutations, while 6 patients had non-classic mutations also considered pathogenic (in 1 patient with two additional mutations of unknown significance) and 1 patient had a *BRAF* mutation of unknown significance. The most common *PIK3CA* mutation in *BRAF* mutated cases were at H1047 but the predominance of H1047 position mutations compared to other classic hotspot positions was less pronounced than in TCGA. Mutations in the hotspot amino acid positions E542 (4 patients), E545 (3 patients), Q546 (6 patients), and H1047 (6 patients) of *PIK3CA* were present in 19 of 35 *BRAF*/*PIK3CA* double mutant patients, and 12 additional patients had pathogenic non-hotspot *PIK3CA* mutations. Four patients had mutations of unknown significance.

Compared with colorectal cancer patients without *BRAF* mutations, *BRAF* mutant colorectal cancer patients in the TCGA cohort presented at a more advanced age (Table 1). This was true independently of whether they had concomitant *PIK3CA* mutations. However, no significant differences were observed in age at presentation of *BRAF* mutant colorectal cancer patients in the DFCI cohort (Table 2). *BRAF* mutant patients and even more so *BRAF*/*PIK3CA* double mutant colorectal cancers in TCGA tended to be of earlier stage (stages I and II) than *BRAF* wild type disease (*p* = 0.003, Table 1). In the double mutant group, 86.4% of patients presented with stage I or II cancers. This was also observed in the DFCI cohort, where *BRAF* mutant colorectal cancers with or without concomitant *PIK3CA* mutations presented less often as stage III or IV than colorectal cancer with both genes on a wild type configuration (Table 2). At odds with TCGA, the group with mutant *PIK3CA* and wild type *BRAF* had similar prevalence of stage III and stage IV presentation, compared with the double mutant group in the DFCI cohort. *BRAF* mutant cancers were located almost exclusively in the colon and rarely in the rectum. Rectal cancers constituted 25.9% and 22.2% of cases in the whole TCGA and DFCI cohorts, respectively.

*BRAF* mutant cancers were more commonly MSI high or POLE positive. Compared with *BRAF* mutant/*PIK3CA* wild type cancers, double mutant cancers were even more often MSI or POLE positive (Table 3). Consistent with the high prevalence of MSI and POLE subtype, *BRAF*/*PIK3CA* double mutant colorectal cancers in TCGA had a high Tumor Mutation Burden (TMB) above 200 in 86.4% of cases (Table 3). *BRAF* mutant cancers with wild type *PIK3CA* had TMB above 200 in 70% of cases. In the DFCI cohort, 77.1% of *BRAF*/*PIK3CA* double mutant colorectal cancers and 65.2% of *BRAF* mutant cancers with wild type *PIK3CA* have a TMB above 200 (Table 4). In contrast, in the TCGA cohort, colorectal cancers with wild type *BRAF* had TMB above 200 in 16.8% of cases when *PIK3CA* was mutant and in 7.3% of cases when *PIK3CA* was wild type (Table 3). In the DFCI cohort, colorectal cancers with wild type *BRAF* had TMB above 200 in 35% of cases when *PIK3CA* was mutant and in 18.5% of cases when *PIK3CA* was also wild type (Table 4). 

**Figure 1:**
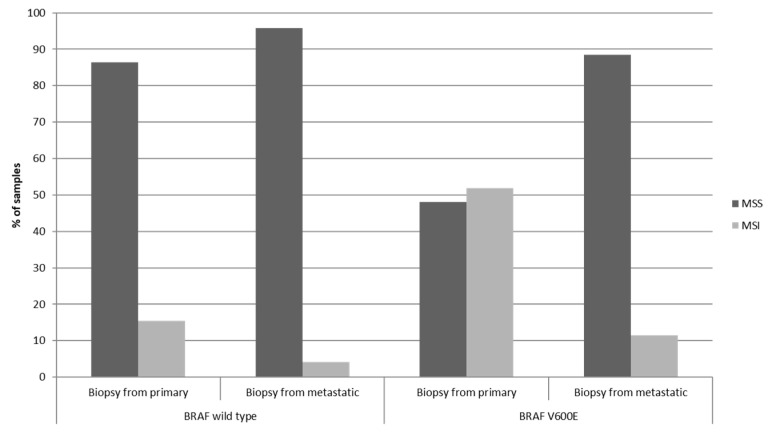
Prevalence of MSI in colorectal cancer biopsies from the primary tumor or from metastatic sites depending on the presence of *BRAF* V600E mutations. Data are from the MSKCC cohort in which all patients had metastatic colorectal cancer. Samples with non-V600E *BRAF* mutations were excluded from this analysis. MSI: Microsatellite Instability high; MSS: Microsatellite Stable.

In the metastatic colon cancer study cohort published by MSKCC, with 1099 patients, *BRAF* mutations were present in 10.8% of patients. Among these patients, about one third or 3.4% of the whole cohort had *BRAF* and *PIK3CA* double mutant cancers. Patients in this study, although all with metastatic disease, had genomic evaluation either from a metastatic site biopsy (n = 522) or from the primary tumor (n = 596, a few patients had biopsies from both sites). *BRAF* mutated cancers evaluated from primary tumor biopsy showed MSI high in 51.9% of samples while in *BRAF* mutated cancers evaluated from metastatic site biopsies showed MSI high in 11.5% of samples (Fisher’s exact test *p* = 0.02, Figure 1). These data suggest that, in MSI high *BRAF* mutated colorectal cancers, metastatic disease develops from clones that have become MSS or had been MSS from the beginning.

*BRAF* mutant colorectal cancers show a higher prevalence of chromosomal stability as determined by low Aneuploidy Score (AS) and low Fraction of Genome Altered (FGA) score. *BRAF*/*PIK3CA* double mutant cancers had higher rates of chromosomal stability than *BRAF* mutant cancers with wild type *PIK3CA* (Table 3). On the other hand, *BRAF* wild type colorectal cancers show higher rates of chromosomal instability (CIN) as determined by higher AS and FGA scores. The most frequent amplified locus in colorectal cancer at chromosome 20q11.21 is observed exclusively in cancers with wild type *BRAF*, with or without mutations in *PIK3CA* (in about 10% of cases in TCGA, not shown). The CpG Island Methylator Phenotype (CIMP) is more prevalent in *BRAF* mutant cancers than in *BRAF* wild type cancers (Table 4).

**Figure 2:**
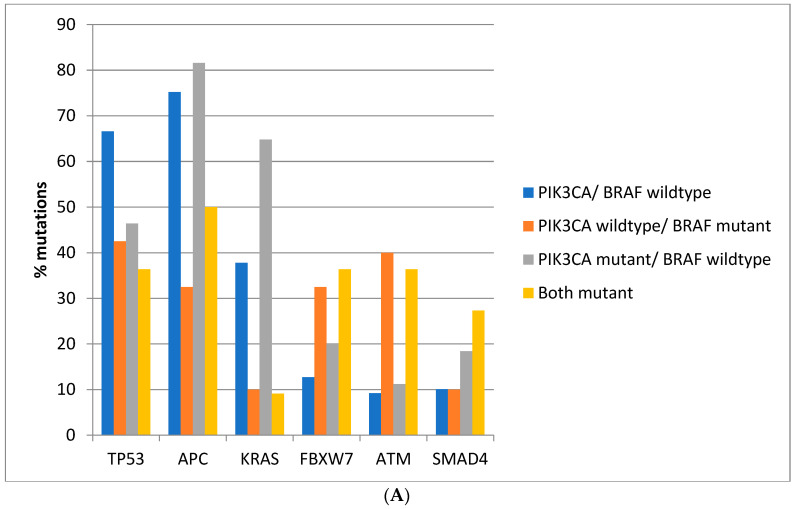

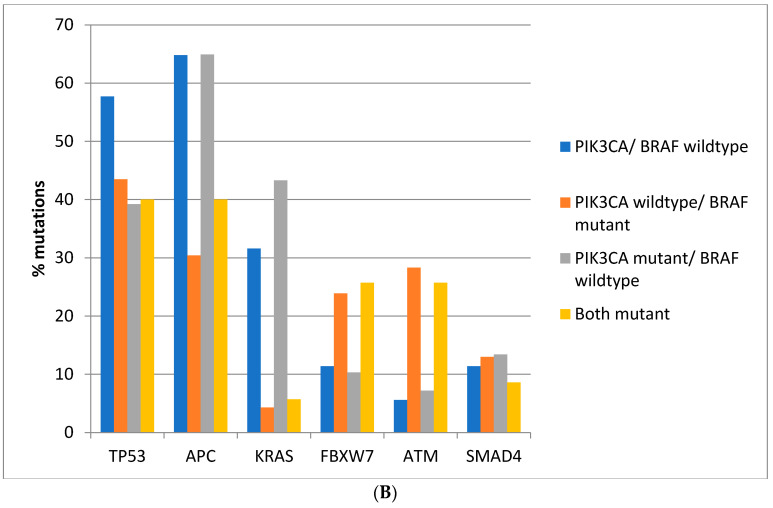
Prevalence of mutations in cancer-associated genes frequently mutated in colorectal cancer in colorectal cancer patients with or without *BRAF* and *PIK3CA* mutations. (**A**) TCGA cohort. (**B**) DFCI cohort.

Regarding the prevalence of mutations in other commonly mutated oncogenes and tumor suppressors, there are significant differences between colorectal cancers depending on the presence of *BRAF* and *PIK3CA* mutations. Mutations in the tumor suppressor *TP53* are more prevalent in colorectal cancers of the TCGA cohort with *BRAF* and *PIK3CA* wild type (66.6%) compared with cancers with either or both *BRAF* and *PIK3CA* mutated, where mutations in *TP53* are encountered in 36.4–46.4% of cases (Fisher’s exact test *p* < 0.0001, Figure 2A). Similarly, in the DFCI cohort, the prevalence of *TP53* mutations was 57.7% in *BRAF* and *PIK3CA* wild type cancers and 39.2–43.5% in cancers with either or both *BRAF* and *PIK3CA* mutated (Fisher’s exact test *p* < 0.0001, Figure 2B). Mutations in the tumor suppressor *APC* are more prevalent in *BRAF* wild type colorectal cancers independently of the presence or absence of concomitant *PIK3CA* mutations in both cohorts (Fisher’s exact test *p* < 0.0001 for the comparisons in both cohorts, Figure 2A,B). Oncogene *KRAS* mutations are rather rare in *BRAF* mutant colorectal cancers, occurring in 10% of such cancers in TCGA and in about 5% in the DFCI cohort, compared with 37.8% and 31.6% in cancers with *BRAF* and *PIK3CA* wild type and 64.8% and 43.3% of cancers with *BRAF* wild type and *PIK3CA* mutations (Figure 2A,B). Tumor suppressor *FBXW7* and *ATM* mutations are more common in *BRAF* mutant cancers with or without *PIK3CA* mutations than in *BRAF* wild type colorectal cancers (Fisher’s exact test *p* = 0.0004 for *FBXW7* and *p* < 0.0001 for *ATM* in TCGA and Fisher’s exact test *p* = 0.0003 for *FBXW7* and *p* < 0.0001 for *ATM* in the DFCI cohort, Figure 2). *SMAD4* mutations show a higher prevalence in *BRAF*/*PIK3CA* double mutant cancers of the TCGA cohort but not in cancers with *BRAF* mutations without *PIK3CA* mutations. In addition, no significant differences in *SMAD4* mutations’ prevalence were observed in the DFCI cohort. 

**Figure 3:**
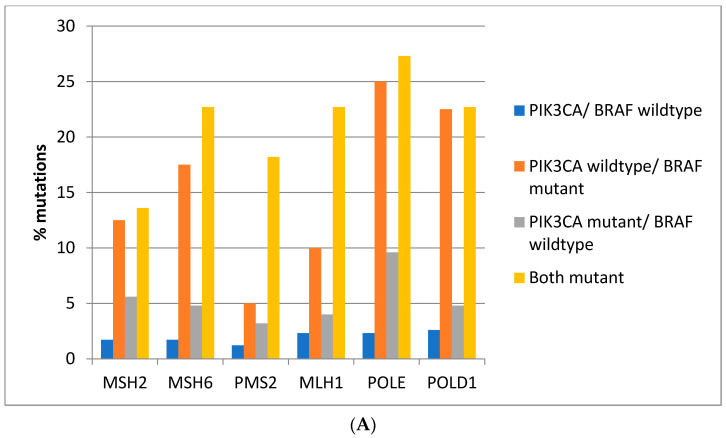

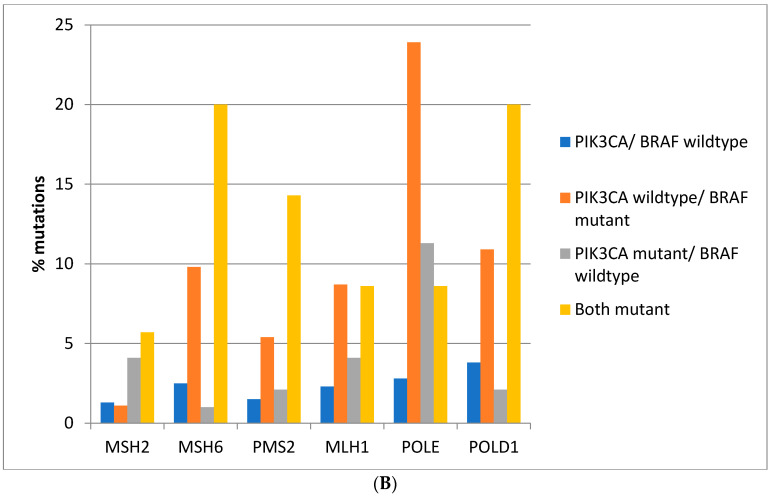
Prevalence of mutations in MMR-associated genes and in the genes encoding for proof-reading polymerases epsilon and delta in colorectal cancer patients with or without *BRAF* and *PIK3CA* mutations. (**A**) TCGA cohort. (**B**) DFCI cohort.

Mutations in the genes associated with MMR (*MSH2*, *MSH6*, *PMS2*, and *MLH1*) and those encoding for proof-reading polymerases epsilon (*POLE*) and delta (*POLD1*) show higher mutation rates in *BRAF*/*PIK3CA* double mutant colorectal cancers while cancers with *BRAF* mutations and *PIK3CA* wild type display similarly high mutation rates except for *PMS2* and *MLH1* which show significantly lower mutation rates (Figure 3A). Consistently, in the DFCI cohort, the highest prevalence of MMR/proof-reading polymerases mutations is in double *BRAF* and *PIK3CA* mutant colorectal cancers with the exception of *POLE* which shows the highest mutation prevalence in *BRAF* mutant cancers without *PIK3CA* mutations (Figure 3B).

The majority of mutations observed in TCGA and DFCI cohorts in the three most frequently mutated cancer-related genes in colorectal cancer, *TP53*, *KRAS*, and *APC* are oncogenic or likely oncogenic. In the colorectal cancer TCGA cohort, 99.1% of *TP53* mutations, 98.7% of *KRAS* mutations, and 89.7% of *APC* mutations are deemed oncogenic or likely oncogenic by the OncoKB database. Similarly, in the DFCI cohort, 98.6%, 97.2%, and 89.2% of mutations in *TP53*, *KRAS*, and *APC* are oncogenic or likely oncogenic. The functional implications of other frequent mutations in colorectal cancer, occurring in more than 10% of cases, are shown in Table 5. The oncogenicity or likely oncogenicity of these mutations varies from 15.3% for atypical cadherin gene *FAT1* to 84.1% for ubiquitin ligase gene *FBXW7*, in the entire cohort, while the rest of the observed mutations are variants of unknown significance. Although the number of mutations in individual genes are low, when colorectal cancers are categorized according to *BRAF* and *PIK3CA* mutations, the prevalence of oncogenic or likely oncogenic mutations in these genes as a whole is higher in colorectal cancers with both *BRAF* and *PIK3CA* wild type (63.4%) than in double mutant cancers (47.3%) or cancers with *BRAF* mutated and *PIK3CA* wild type (41.7%) and with *PIK3CA* mutant and *BRAF* wild type (49.8%, χ^2^ *p* < 0.001, Table 5). However, in the DFCI cohort, although double *BRAF* and *PIK3CA* wild type colorectal cancers have a numerically higher prevalence of oncogenic/likely oncogenic mutations in commonly mutated cancer-associated genes (53.8%), the differences from the three other groups (*BRAF* or *PIK3CA* mutated or both mutated) were small and borderline statistically insignificant (χ^2^ *p* = 0.051, Table 6).

Mutations in genes associated with MMR and encoding for proof-reading polymerases *POLE* and *POLD1* are designated as oncogenic or likely oncogenic in 20–25% of cases and show no significant differences in their oncogenic functional status in the four groups according to *BRAF* and *PIK3CA* mutations in either TCGA or DFCI cohorts (Table 7 and Table 8).

**Figure 4:**
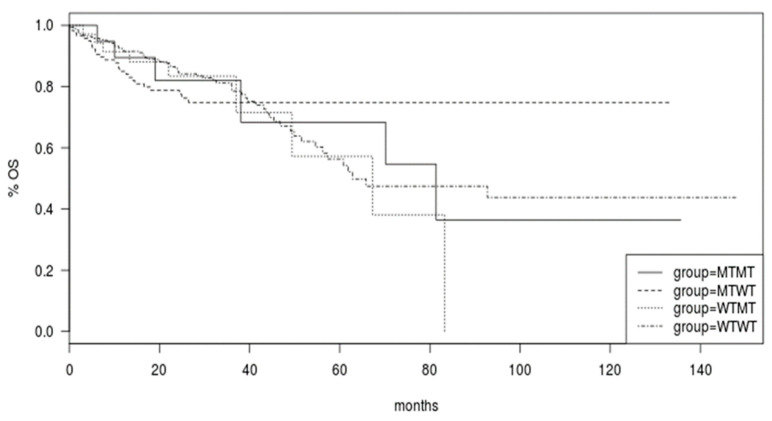
Overall Survival (OS) of patients in the colorectal cancer cohort of TCGA according to the mutational status of *BRAF* and *PIK3CA* genes. Group WTWT: both genes wild type; group WTMT: *PIK3CA* wild type/*BRAF* mutant; group MTWT: *PIK3CA* mutant/*BRAF* wild type; MTMT: both genes mutated.

Survival of colorectal cancer patients of the TCGA was not different in the groups with *BRAF* and *PIK3CA* mutated or both genes mutated compared with cancers with both genes being wild type (Log Rank *p* = 0.9, Figure 4).

## 4. Discussion

*BRAF* is an oncogenic serine/threonine kinase which is frequently mutated in various cancers. In colorectal cancers, the prevalence of *BRAF* mutations is 8–12% [26]. The majority of *BRAF* mutations result in the classical V600E position substitution. In a smaller number of cases, mutations resulting in substitutions on other amino acid positions of the protein, including at positions G469, G496, K601, L597, D594, G596, and G466, are present [27]. While V600E substitutions enable the protein to signal autonomously as a monomer and activate the downstream ERK cascade, alternative substitutions require *BRAF* homodimerization or heterodimerization with other RAF proteins. At odds with canonical V600E mutations, some alternative site mutations retain dependence to upstream KRAS signals for activation [27].

*KRAS* mutations are more common than *BRAF* mutations and are present in 30–40% of colorectal cancers [18]. *KRAS* codon 12 or 13 mutations are mutually exclusive with *BRAF* V600 mutations. For example, in a TCGA colorectal study with 534 analyzed cases only one sample had a concomitant *KRAS* G12D substitution with a *BRAF* V600E substitution. In the DFCI series, only one of the six *KRAS* mutations occurring in samples with *BRAF* mutations was a classic G13D substitution and the concomitant *BRAF* mutation was a G469E substitution. In contrast to *KRAS* mutations, mutations of the gene encoding for the catalytic sub-unit alpha of kinase PI3K, *PIK3CA*, which are common in colorectal cancer are not mutually exclusive with *BRAF* mutations. Among patients with *BRAF* mutations, 35.5% of patients in TCGA and 27.5% of patients in the DFCI cohort had concomitant *PIK3CA* mutations. This prevalence is higher than the overall prevalence of *PIK3CA* mutations in the two series (27.5% in TCGA and 21.3% in DFCI).

In the current work, using data from TCGA and the DFCI cohorts, it is shown that double mutant *BRAF*/*PIK3CA* colorectal cancers and *BRAF* mutant colorectal cancers without *PIK3CA* mutations, which represent 4.1% and 7.5% of cases in TCGA and 5.7% and 14.9% of cases in the DFCI cohort, respectively, differ in their clinical and genomic characteristics from the groups of patients without *BRAF* mutations. *BRAF* mutations are the defining molecular alteration that links double mutant *BRAF*/*PIK3CA* colorectal cancers with MSI, a higher TMB, high CIMP, and low CIN, given that cancers with *BRAF* mutant/*PIK3CA* wild type cancers have similar rates of these characteristics without statistically significant differences compared to double mutants. Although not all *BRAF* mutated colorectal cancers are MSI high, the association of *BRAF* mutations with MSI and CIMP has been previously reported and MSI is commonly the result of MLH1 suppression through promoter methylation in cancers with *BRAF* mutations [28]. About 60% of sporadic MSI high colorectal cancers exhibit *BRAF* mutations [29]. However, the presence of *PIK3CA* mutations in *BRAF* mutated colorectal cancers leads to a numeric increase in the prevalence of MSI (from 59.5% to 68.2% in TCGA and from 47.1% to 62.9% in the DFCI cohort). In contrast, colorectal cancers with *PIK3CA* mutations but without *BRAF* mutations have statistically significant lower MSI, TMB, and CIMP rates and higher CIN rates compared with double mutants.

Despite different platforms used in the two studied cohorts, molecular alterations observed are consistent between them, with few differences. One such difference is in the mutation rate of tumor suppressor SMAD4, which showed a higher prevalence in double mutant cancers in TCGA but not in the DFCI cohort, where all four groups had similar *SMAD4* mutation rates. SMAD4 loss of function has been implicated in the serrated pathway of colorectal carcinogenesis where *BRAF* mutations are also present [30]. In a mouse model of colorectal carcinogenesis in vivo, *BRAF* V600E mutations in intestinal stem cells promote differentiation and require inactivation of SMAD4 or of intestinal differentiation transcription factor CDX2 for efficient tumor formation [31]. SMAD4 inactivation has been suggested as an important molecular event in the group of *BRAF* mutated serrated carcinomas that are microsatellite stable [30]. However, mutations of *SMAD4* occur only in a minority of *BRAF* mutated colorectal cancers and accumulation of alternative lesions may be needed to advance *BRAF*-associated carcinogenesis. Indeed, activation of the WNT/β-catenin pathway is required even in the presence of SMAD4 inactivation in *BRAF*-associated colorectal cancers [30]. Given that the prevalence of *APC* mutations is shown to be lower in *BRAF* mutated colorectal cancers, alternative modes of pathway activation are at play and may involve inhibition of kinase GSK3 through PI3K/AKT signaling [32,33]. Thus, concomitant *PIK3CA* mutations could provide the required WNT/β-catenin pathway activation in *BRAF* mutated cancers.

*BRAF* mutations are used as biomarkers of therapy guidance in metastatic colorectal cancer based on the results of the phase III randomized BEACON trial [5]. This trial established that the combination of the *BRAF* inhibitor encorafenib with EGFR monoclonal antibody cetuximab was superior to chemotherapy in pretreated patients with metastatic colorectal cancer bearing *BRAF* V600E mutations. The study included a triplet arm with the MEK inhibitor binimetinib in addition to encorafenib and cetuximab which had a higher overall response rate than the doublet arm (26.8% versus 19.5% in the doublet arm). Despite that, median overall survival (OS) was 9.3 months in both targeted therapies arms [6]. Median OS was 5.9 months in the control arm treated with chemotherapy. Thus, the encorafenib with cetuximab doublet is the preferred second line treatment for metastatic colorectal cancers with V600E *BRAF* mutations. Besides showing that MEK inhibition with binimetinib has no benefit for OS, the BEACON results establish that pretreated patients with *BRAF* mutations have short survivals even with the improved outcomes provided by the targeted treatment. It is worth noting that only 10% of the patients that participated in BEACON had MSI high cancers compared with a prevalence of MSI exceeding 50% in *BRAF* mutant colorectal cancers of the TCGA and DFCI cohorts. Similarly, in a “real life” cohort of *BRAF* mutant metastatic colorectal cancers treated with targeted therapies as used in the BEACON trial in several Italian centers, the prevalence of MSI or Mismatch Repair deficiency was 15% [34]. This suggests that MSS cancers are enriched in the metastatic setting and that MSI-associated *BRAF* mutated cancers progress less often to a metastatic stage. Consistently, data from a metastatic cohort, presented here, confirm that *BRAF* mutations in biopsies from the patients’ primary tumor display MSI high in 37.8% of cases while biopsies from metastatic sites in similar *BRAF* mutated cancer patients from the same cohort showed MSI high in 11.5% of cases. The prevalence of high TMB, in the range associated with putative responses to immune checkpoint inhibitors, is also lower in metastatic *BRAF* mutated cancers, when evaluated from biopsy samples of metastatic sites compared with samples from biopsies of the primary tumor [20]. In contrast, MSI high, *BRAF* mutated cancers have in general a better prognosis [35,36]. In patients with stage III colorectal cancers who participated in the NCCTG N0147 trial, event-free survival (EFS) of the group with *BRAF* mutations and MMR deficiency was similar to cancers with MMR proficiency and no *BRAF* or *KRAS* mutations, while EFS of proficient for MMR cancers with *BRAF* mutations was inferior [35]. OS is also inferior in stage III *BRAF* mutant MSS colorectal cancers compared to MSS colorectal cancers with wild type *BRAF*, but no difference dependent on *BRAF* status is present in MSI high cancers [37]. In addition, in metastatic colorectal cancer patients participating in four trials, *BRAF* mutations were associated with worse PFS and OS in MMR proficient but not in MMR deficient cancers [36].

*BRAF* mutant cancers have been divided in two subsets, based on unsupervised genomic clustering, that do not correlate with their MSI status or *PIK3CA* mutation status [38]. One of the sub-types called BM1 displays activation of KRAS/AKT and mTOR/protein translation pathways as well as features of epithelial to mesenchymal transition. The other sub-type BM2, which is more frequent, presents deregulation of cell cycle checkpoints as the main feature [38]. Despite the lack of association of this clustering directly with the presence of concomitant mutations in *PIK3CA*, the fact that the BM1 sub-type includes activation of the pathway among its defining characteristics suggests that this activation, independently of the specific nature of the underlying molecular lesion(s) producing it, is important in the pathogenesis of this *BRAF* mutated sub-set. Moreover, in some cancers of the BM2 sub-type the presence of *PIK3CA* mutations is not sufficient to activate the KRAS/AKT and mTOR/protein translation pathways. Despite the key function of PI3K/AKT/mTOR activity in a sub-set of *BRAF* mutated colorectal cancers, few trials have attempted to target the pathway in these cancers or to systematically exploit therapeutically the sub-set with concomitant *BRAF* and *PIK3CA* mutations. A phase Ib trial of encorafenib, cetuximab with or without alpelisib in 54 metastatic *BRAF* mutated colorectal cancer patients showed no significant difference in median PFS which was 3.7 months with the dual combination and 4.2 moths with the addition of alpelisib [39]. A next generation sequence analysis which was performed in a subset of 21 patients (13 patients in the doublet arm and 8 patients in the triplet arm) showed that 3 of 7 patients (2 in the triplet arm and 1 patient in the doublet arm) with median PFS around or longer than 6 months had concomitant mutations in *PIK3CA* while none of 14 patients with median PFS shorter than 6 months had such mutations [39].

An additional key observation of the current report is that *BRAF* mutated colorectal cancers with or without *PIK3CA* mutations display lower prevalence of APC mutations than colorectal cancers with wild type *BRAF*. Moreover, *BRAF* mutated colorectal cancers with or without *PIK3CA* mutations, as well as *PIK3CA* mutated cancers without *BRAF* mutations possess lower mutation rates of *TP53*, suggesting that activation of either or both oncogenes decrease pressure for disabling of *TP53* in colorectal cancer cells. A negative correlation of *PIK3CA* mutations and *TP53* mutations was also observed in a recently published series, which did not examine *BRAF* mutations [40]. In contrast to *APC* and *TP53*, the prevalence of mutations in tumor suppressors *FBXW7* and *ATM* is higher in *BRAF* mutated cancers. A high prevalence of *ATM* mutations is observed in *BRAF* mutated colorectal cancers in both TCGA and DFCI cohorts. In TCGA, *ATM* mutations are present in 40% of cases with *PIK3CA* wild type and in 36.4% of cases with *PIK3CA* mutations. In the DFCI cohort, *ATM* mutations are present in 28.3% of cases with *PIK3CA* wild type and in 25.7% of cases with *PIK3CA* mutations. The prevalence of *ATM* mutations in the entire TCGA and DFCI cohorts is 13.1 % and 10.3%, respectively. Other series have shown an *ATM* mutation prevalence of 15% in metastatic colorectal cancer but no increased prevalence in *BRAF* mutated cases compared to *BRAF* wild type metastatic colorectal cancers [41]. This suggests that *ATM* mutations in *BRAF* mutated colorectal cancers are associated with a better prognosis sub-group such as MSI high. Indeed, 21 of 24 *BRAF* and *ATM* mutated colorectal cancers (87.5%) in TCGA are MSI or POLE subtype while among *BRAF* mutated, in *ATM* wild type cancers only 55.3% belong to these subtypes (Fisher’s exact test *p* = 0.01). In total, 20 of the 24 *ATM* mutations (83.3%) in *BRAF* mutated colorectal cancers are categorized as likely oncogenic. In addition to *ATM*, other genes related to DNA damage response (DDR) are mutated in smaller percentages of colorectal cancers and show a predilection for *BRAF* mutated cancers. Mutations in DDR involved genes leading to homologous recombination defects sensitized to PARP inhibitors [42]. In colorectal cancer, a phase II trial examining the PARP inhibitor olaparib as monotherapy in pretreated metastatic colorectal cancer patients showed no responses in either MSI or MSS disease [43]. A phase I trial of olaparib in combination with irinotecan in unselected metastatic colorectal cancer patients showed also no responses and 9 of 25 patients had stable disease as the best response [44]. The olaparib/irinotecan combination was used in a heavily pretreated metastatic colorectal cancer patient with an *ATM* mutation who obtained stable disease for 3 months with a longer clinical and serologic markers improvement [45]. Although PARP inhibitors in unselected colorectal cancer patients seem to have minimal activity, study of specific molecular subsets and of combinations with other targeted treatments deserve consideration. Combinations with *BRAF* inhibitors in *BRAF* mutated cancers with *ATM* mutations or other homologous recombination defects could be a prime target.

In conclusion, the current study shows that the complex landscape of *BRAF* mutated colorectal cancer with or without concomitant *PIK3CA* mutations offers several leads for therapeutic targeting to improve outcomes of this subset of metastatic cancer patients associated with adverse survival. Newer *BRAF* inhibitors in development which avoid the paradoxical activation of wild type *BRAF* mediated by current inhibitors in use are also expected to advance therapeutics of these difficult to treat, resistant cancers [46].

## Figures and Tables

**Table 1:** Characteristics of colorectal cancers with and without *PIK3CA* and *BRAF* mutations in the TCGA cohort. Two patients are missing demographic data. Analysis for *PIK3CA* and *BRAF* mutations was performed in 534 patient samples. NA: not available. The *p* values presented in the last column refer to Fisher’s exact test except if stated otherwise. For each categorical characteristic, three comparisons are presented. The first includes all four groups (group with both *PIK3CA* and *BRAF* wild type, group with *PIK3CA* wild type and *BRAF* mutant, group with *PIK3CA* mutant and *BRAF* wild type, and group with both *PIK3CA* and *BRAF* mutant), the second comparison is between double mutant cancers and cancers with *BRAF* mutations and wild type *PIK3CA*. The third comparison is between double mutant cancers and cancers with *PIK3CA* mutated and *BRAF* wild type. For cancer stage comparisons in the four mutation groups, early stages (I and II) and advanced stages (III and IV) were grouped together in the statistical analysis. The comparisons of various characteristics of interest involving all four mutation groups were performed with the χ^2^ test. Comparisons involving two groups were performed with the Fisher’s exact test when the characteristic of interest had 2 categories and with the χ^2^ test when the characteristic of interest had more than 2 categories.

Characteristic	Entire Cohort (n = 594)	*PIK3CA* and *BRAF* Wild Type (n = 347)	*PIK3CA* Wild Type/*BRAF* Mutant (n = 40)	*PIK3CA* Mutant/*BRAF* Wild Type (n = 125)	Both Mutant (n = 22)	*p*
Age (mean ± SD)	66.1 ± 13.4	64.9 ± 12.9	70.4 ± 14.4	66 ± 12.3	69.7 ± 12.9	0.03 (ANOVA)
Age						
≤65 years old	260 (43.9)	161 (46.5)	13 (32.5)	60 (48.4)	6 (27.3)	0.1 (χ^2^)
>65 years old	332 (56.1)	185 (53.5)	27 (67.5)	64 (51.6)	16 (72.7)	0.7
NA	2	1	0	1	0	0.1
Sex						
Male	312 (52.7)	181 (52.3)	17 (42.5)	70 (56.5)	9 (40.9)	0.31 (χ^2^)
Female	280 (47.3)	165 (47.7)	23 (57.5)	54 (43.5)	13 (59.1)	1
NA	2	1	0	1	0	0.24
Stage						
I	104 (17.9)	65 (19.1)	4 (10)	19 (16.1)	7 (31.8)	I–II versus III–IV
II	220 (37.9)	113 (33.2)	23 (57.5)	53 (44.9)	12 (54.6)	0.003 (χ^2^)
III	170 (29.3)	105 (30.9)	10 (25)	33 (28)	3 (13.6)	0.13
IV	86 (14.8)	57 (16.8)	3 (7.5)	13 (11)	0	0.02
NA	14	7	0	7	0	
Location primary						
Colon	436 (74.1)	229 (67)	37 (92.5)	105 (84.7)	20 (90.9)	<0.0001 (χ^2^)
Rectal	152 (25.9)	113 (33)	3 (7.5)	19 (15.3)	2 (9.1)	1
NA	6	5	0	1	0	0.7

**Table 2:** Characteristics of colorectal cancers with *BRAF* and *PIK3CA* mutations in the DFCI cohort. NA: not available. Right cancers include cecum to transverse column and left cancers include those located from the splenic flexure to rectum. The *p* values presented in the last column refer to Fisher’s exact test except if stated otherwise. For each categorical characteristic, three comparisons are presented. The first includes all four groups (group with both *PIK3CA* and *BRAF* wild type, group with *PIK3CA* wild type and *BRAF* mutant, group with *PIK3CA* mutant and *BRAF* wild type, and group with both *PIK3CA* and *BRAF* mutant), the second comparison is between double mutant cancers and cancers with *BRAF* mutations and wild type *PIK3CA*. The third comparison is between double mutant cancers and cancers with *PIK3CA* mutated and *BRAF* wild type. For cancer stage comparisons in the four mutation groups, early stages (I and II) and advanced stages (III and IV) were grouped together in the statistical analysis. For the primary tumor location comparisons in the four groups, right colon cancers (cecum and ascending to transverse) and left colon cancers (splenic flexure to sigmoid and rectal) were grouped together in the statistical analysis. The comparisons of various characteristics of interest involving all four mutation groups were performed with the χ^2^ test. Comparisons involving two groups were performed with the Fisher’s exact test when the characteristic of interest had 2 categories and with the χ^2^ test when the characteristic of interest had more than 2 categories.

Characteristic	Entire Cohort (n = 619)	*PIK3CA* and *BRAF* Wild Type (n = 395)	*PIK3CA* Wild Type/*BRAF* Mutant (n = 92)	*PIK3CA* Mutant/*BRAF* Wild Type (n = 97)	Both Mutant (n = 35)	*p*
Age (mean ± SD)	70.7 ± 8.6	70.3 ± 8.6	71.9 ± 7.5	71 ± 9.4	71.4 ± 7.8	0.29 (ANOVA)
Age						
≤65 years old	160 (25.9)	114 (28.9)	17 (18.7)	23 (23.7)	6 (17.1)	0.11 (χ^2^)
>65 years old	457 (74.1)	280 (71.1)	74 (81.3)	74 (76.3)	29 (82.9)	1
NA	2	1	1			0.48
Sex						
Male	239 (38.6)	160 (40.5)	20 (21.7)	44 (45.4)	15 (42.9)	0.003 (χ^2^)
Female	380 (61.4)	235 (59.5)	72 (78.3)	53 (54.6)	20 (57.1)	0.02
Stage						0.84
I	152 (27)	101 (28.5)	18 (20.2)	27 (32.2)	6 (17.1)	I–II versus III–IV
II	187 (33.2)	98 (27.6)	37 (41.6)	33 (39.3)	19 (54.3)	0.03 (χ^2^)
III	159 (28.2)	114 (32.1)	22 (24.7)	17 (20.2)	6 (17.1)	0.4
IV	65 (11.6)	42 (11.8)	12 (13.5)	7 (8.3)	4 (11.4)	1
NA	56	40	3	13		
Location primary						
Cecum	114 (18.4)	65 (16.5)	11 (12)	27 (27.8)	11 (31.4)	Right versus Left
Ascending to transverse	201 (32.5)	86 (21.8)	65 (70.7)	31 (32)	19 (54.3)	<0.0001 (χ^2^)
Splenic flexure to sigmoid	166 (26.9)	121(30.7)	12 (13)	28 (28.9)	5 (14.3)	0.79
Rectal	137 (22.2)	122 (31)	4 (4.3)	11 (11.3)	0	0.006
NA	1	1				

**Table 3:** Prevalence of cases according to Tumor Mutation Burden (TMB), Aneuploidy Score (AS), and Fraction Genome Altered (FGA) in the groups with or without *BRAF* and *PIK3CA* mutations in TCGA. Analysis for *PIK3CA* and *BRAF* mutations was performed in 534 samples. GS: Genomic Stable; CIN: Chromosomal Instability; MSI: Microsatellite Instability; NA: not available. The *p* values presented in the last column refer to the χ^2^ test. For each categorical characteristic, three comparisons are presented. The first comparison includes all four groups (group with both *PIK3CA* and *BRAF* wild type, group with *PIK3CA* wild type and *BRAF* mutant, group with *PIK3CA* mutant and *BRAF* wild type, and group with both *PIK3CA* and *BRAF* mutant), the second comparison is between double mutant cancers and cancers with *BRAF* mutations and wild type *PIK3CA*. The third comparison is between double mutant cancers and cancers with *PIK3CA* mutated and *BRAF* wild type.

Characteristic	Entire Cohort (n = 594)	*PIK3CA* and *BRAF* Wild Type (n = 347)	*PIK3CA* Wild Type/*BRAF* Mutant (n = 40)	*PIK3CA* Mutant/*BRAF* Wild Type (n = 125)	Both Mutant (n = 22)	*p*
Subtype						
Colon GS	49 (10.7)	24 (8.2)	3 (8.1)	20 (18.7)	2 (9.1)	GS vs. CIN vs. MSI
Colon CIN	226 (49.2)	159 (54.3)	10 (27)	56 (52.4)	1 (4.5)	<0.0001
Colon MSI	60 (13.1)	15 (5.1)	21 (56.8)	9 (8.4)	15 (68.2)	0.07
Colon POLE	6 (1.3)	0	0	4 (3.7)	2 (9.1)	<0.0001
Rectal GS	9 (2)	6 (2)	0	3 (2.8)	0	
Rectal CIN	102 (22.2)	87 (29.8)	1 (2.7)	14 (13.1)	0	
Rectal MSI	3 (0.6)	1 (0.3)	1 (2.7)	1 (0.9)	0	
Rectal POLE	4 (0.9)	1 (0.3)	1 (2.7)	0	2 (9.1)	
NA	135	54	3	18	0	
TMB						
<100	243 (46)	195 (57.2)	3 (7.5)	44 (35.2)	1 (4.5)	<0.0001
100–200	192 (36.4)	121 (35.5)	9 (22.5)	60 (48)	2 (9.1)	0.2
>200	93 (17.6)	25 (7.3)	28 (70)	21 (16.8)	19 (86.4)	<0.0001
NA	66	6	0	0	0	
AS						
<4	108 (18.4)	30 (8.8)	21 (52.5)	26 (21)	17 (81)	<0.0001
4–24	427 (72.9)	275 (79.9)	19 (47.5)	90 (72.6)	4 (19)	0.05
>24	51 (8.7)	39 (11.3)	0	8 (6.4)	0	<0.0001
NA	8	3	0	1	1	
FGA						
<0.08	118 (20.2)	36 (10.5)	22 (56.4)	28 (23.3)	17 (77.3)	<0.0001
0.08–0.35	316 (54.2)	207 (60.5)	15 (38.5)	67 (55.8)	5 (22.7)	0.16
>0.35	149 (25.6)	99 (29)	2 (5.1)	25 (20.8)	0	<0.0001
NA	11	5	1	5	0	

**Table 4:** MSI status, TMB, CIMP in the DFCI cohort. MSI: Microsatellite Instability; TMB: Tumor Mutation Burden; CIMP: CpG Island Methylator Phenotype; NA: not available. The *p* values presented in the last column refer to Fisher’s exact test except if stated otherwise. For each categorical characteristic, three comparisons are presented. The first comparison includes all four groups (group with both *PIK3CA* and *BRAF* wild type, group with *PIK3CA* wild type and *BRAF* mutant, group with *PIK3CA* mutant and *BRAF* wild type, and group with both *PIK3CA* and *BRAF* mutant), the second comparison is between double mutant cancers and cancers with *BRAF* mutations and wild type *PIK3CA*. The third comparison is between double mutant cancers and cancers with *PIK3CA* mutated and *BRAF* wild type.

Characteristic	Entire Cohort (n = 619)	*PIK3CA* and *BRAF* Wild Type (n = 395)	*PIK3CA* Wild Type/*BRAF* Mutant (n = 92)	*PIK3CA* Mutant/*BRAF* Wild Type (n = 97)	Both Mutant (n = 35)	*p*
Subtype						
MSI high	91 (17.2)	23 (6.7)	33 (47.1)	13 (15.9)	22 (62.9)	<0.0001 (χ^2^)
MSS	438 (82.8)	319 (93.3)	37 (52.9)	69 (84.1)	13 (37.1)	0.15
NA	90	53	22	15		<0.0001
TMB						
<100	140 (22.6)	117 (29.6)	5 (5.4)	15 (15.5)	3 (8.6)	<0.0001 (χ^2^)
100–200	285 (46)	205 (51.9)	27 (29.4)	48 (49.5)	5 (14.3)	0.28 (χ^2^)
>200	194 (31.4)	73 (18.5)	60 (65.2)	34 (35)	27 (77.1)	<0.0001 (χ^2^)
CIMP						
High	95 (19)	18 (5.6)	46 (65.7)	9 (11.7)	22 (73.3)	<0.0001 (χ^2^)
0—low	405 (81)	305 (94.4)	24 (34.3)	68 (88.3)	8 (26.7)	0.49
NA	119	72	22	20	5	<0.0001

**Table 5:** Prevalence of functionally annotated mutations as oncogenic/probably oncogenic in commonly mutated (more than 10% of cases in the whole cohort) cancer-associated genes in TCGA. The nominator in each case represents number of oncogenic or probably oncogenic mutations and the denominator represents the total number of mutations of each gene in each group.

Mutation	Entire Cohort (n = 594)	*PIK3CA* and *BRAF* Wild Type (n = 347)	*PIK3CA* Wild Type/*BRAF* Mutant (n = 40)	*PIK3CA* Mutant/*BRAF* Wild Type (n = 125)	Both Mutant (n = 22)	*p*
*TP53*	329/332 (99.1%)	243/244 (99.6%)	18/18 (100%)	59/59 (100%)	9/11 (81.8%)	
*APC*	573/639 (89.7%)	377/400 (94.2%)	18/26 (69.2%)	157/178 (88.2%)	21/35 (60%)	
*KRAS*	220/223 (98.7%)	132/134 (98.5%)	3/4 (75%)	82/82 (100%)	3/3 (100%)	
*FBXW7*	90/107 (84.1%)	42/49 (85.7%)	11/16 (68.8%)	27/31 (87.1%)	10/11 (90.9%)	
*ATM*	53/107 (49.5%)	24/37 (64.7%)	12/29 (41.4%)	8/25 (32%)	9/16 (56.3%)	
*SMAD4*	57/80 (71.3%)	28/40 (70%)	2/4 (50%)	19/26 (73.1%)	8/10 (80%)	
*AMER1*	52/72 (72.2%)	26/31 (83.9%)	4/10 (40%)	17/22 (77.3%)	5/9 (55.6%)	
*SOX9*	54/73 (74%)	27/35 (77.1%)	6/10 (60%)	20/27 (74.1%)	1/1 (100%)	
*KMT2D*	32/90 (35.6%)	10/23 (43.5%)	12/21 (57.1%)	5/27 (18.5%)	5/19 (26.3%)	
*ARID1A*	46/65 (70.8%)	16/21 (76.2%)	5/11 (45.5%)	13/18 (72.2%)	12/15 (80%)	
*KMT2B*	24/74 (32.4)	8/22 (36.4%)	5/18 (27.8%)	3/20 (15%)	8/14 (57.1%)	
*TCF7L2*	40/67 (59.7%)	23/34 (67.6%)	2/3 (66.7%)	13/26 (50%)	2/4 (50%)	
*FAT1*	15/98 (15.3%)	4/22 (18.2%)	4/26 (15.4%)	2/22 (9.1%)	5/29 (17.2%)	
*KMT2C*	23/84 (27.4%)	7/25 (28%)	7/20 (35%)	5/21 (23.8%)	4/18 (22.2%)	
Total	486/918 (52.9%)	215/339 (63.4%)	70/168 (41.7%)	132/265 (49.8%)	69/146 (47.3%)	0.001 (χ^2^)

**Table 6:** Prevalence of functionally annotated mutations as oncogenic/probably oncogenic in DFCI. The nominator in each case represents number of oncogenic or probably oncogenic mutations and the denominator represents the total number of mutations of each gene in each group.

Mutation	Entire Cohort (n = 619)	*PIK3CA* and *BRAF* Wild Type (n = 395)	*PIK3CA* Wild Type/*BRAF* Mutant (n = 92)	*PIK3CA* Mutant/*BRAF* Wild Type (n = 97)	Both Mutant (n = 35)	*p*
*TP53*	340/345 (98.6%)	239/240 (99.6%)	47/47 (100%)	40/42 (95.2%)	14/16 (87.5%)	0.001 (χ^2^)
*APC*	446/500 (89.2%)	313/335 (93.5%)	22/36 (61.1%)	94/104 (90.4%)	17/25 (68%)	<0.0001 (χ^2^)
*KRAS*	172/177 (97.2%)	126/128 (98.4%)	1/4 (25%)	43/43 (100%)	2/2 (100%)	<0.0001 (χ^2^)
*FBXW7*	74/102 (72.5%)	39/50 (78%)	19/27 (70.4%)	9/13 (69.2%)	7/12 (58.3%)	
*ATM*	43/81 (53.1%)	12/25 (48%)	19/35 (54.3%)	8/9 (88.9%)	4/12 (33.3%)	
*SMAD4*	56/82 (68.3%)	35/49 (71.4%)	8/14 (57.1%)	12/16 (75%)	1/3 (33.3%)	
*AMER1*	44/55 (80%)	17/22 (77.3%)	2/11 (18.2%)	15/16 (93.7%)	5/6 (83.3%)	
*SOX9*	53/70 (75.7%)	34/37 (91.9%)	10/18 (55.6%)	8/12 (66.7%)	2/3 (66.7%)	
*KMT2D*	29/96 (30.2%)	9/35 (25.7%)	14/35 (40%)	1/14 (7.1%)	5/12 (41.7%)	
*ARID1A*	45/79 (57%)	14/25 (56%)	10/20 (50%)	8/17 (47.1%)	13/17 (76.5%)	
*KMT2B*	16/52 (30.8%)	2/14 (14.3%)	2/13 (15.4%)	4/13 (30.8%)	6/12 (50%)	
*TCF7L2*	20/46 (43.5%)	13/25 (52%)	2/8 (25%)	3/6 (50%)	2/7 (28.6%)	
*FAT1*	14/78 (17.9%)	2/30 (6.7%)	8/25 (32%)	1/9 (11.1%)	3/14 (21.4%)	
*KMT2C*	28/109 (25.7%)	13/46 (28.3%)	4/24 (16.7%)	7/24 (29.2%)	4/15 (26.7%)	
Total	422/850 (49.6%)	190/353 (53.8%)	98/230 (42.6%)	76/148 (51.4%)	52/113 (46%)	0.051 (χ^2^)

**Table 7:** Prevalence of functionally annotated mutations as oncogenic/probably oncogenic in MMR and proof-reading polymerases associated genes in TCGA. The nominator in each case represents number of oncogenic or probably oncogenic mutations and the denominator represents the total number of mutations of each gene in each group.

Mutation	Entire Cohort (n = 594)	*PIK3CA* and *BRAF* Wild Type (n = 347)	*PIK3CA* Wild Type/*BRAF* Mutant (n = 40)	*PIK3CA* Mutant/*BRAF* Wild Type (n = 125)	Both Mutant (n = 22)	*p*
*MSH2*	10/27 (37%)	3/6 (50%)	3/9 (33.3%)	4/8 (50%)	0/4	
*MSH6*	9/29 (31%)	3/6 (50%)	3/8 (37.5%)	3/8 (37.5%)	0/7	
*PMS2*	4/16 (25%)	1/5 (20%)	1/3 (33.3%)	1/4 (25%)	1/4 (25%)	
*MLH1*	13/24 (54.2%)	4/8 (50%)	2/5 (40%)	4/5 (80%)	3/6 (50%)	
*POLE*	10/50 (20%)	1/11 (9.1%)	1/12 (8.3%)	5/19 (26.3%)	3/8 (37.5%)	
*POLD1*	0/32	0/10	0/9	0/7	0/6	
Total	46/178 (25.8%)	12/46 (26.1%)	10/46 (21.7%)	17/51 (33.3%)	7/35 (20%)	0.47 (χ^2^)

**Table 8:** Prevalence of functionally annotated mutations as oncogenic/probably oncogenic of MMR and proof-reading polymerases associated genes in the DFCI cohort. The nominator in each case represents number of oncogenic or probably oncogenic mutations and the denominator represents the total number of mutations of each gene in each group.

Mutation	Entire Cohort (n = 619)	*PIK3CA* and *BRAF* Wild Type (n = 395)	*PIK3CA* Wild Type/*BRAF* Mutant (n = 92)	*PIK3CA* Mutant/*BRAF* Wild Type (n = 97)	Both Mutant (n = 35)	*p*
*MSH2*	4/12 (33.3%)	2/5 (40%)	0/1	2/4 (50%)	0/2	
*MSH6*	12/29 (41.4%)	3/10 (30%)	4/10 (40%)	1/1 (100%)	4/8 (50%)	
*PMS2*	5/19 (26.3%)	3/6 (50%)	1/6 (16.7%)	1/2 (50%)	0/5	
*MLH1*	13/25 (52%)	4/10 (40%)	3/8 (37.5%)	3/4 (75%)	3/3 (100%)	
*POLE*	5/56 (8.9%)	1/11 (9.1%)	2/25 (8%)	1/16 (6.2%)	1/4 (25%)	
*POLD1*	0/37	0/16	0/10	0/2	0/9	
Total	39/178 (21.9%)	13/58 (22.4%)	10/60 (16.7%)	8/29 (27.6%)	8/31 (25.8%)	0.61 (χ^2^)

## Data Availability

No additional data beyond the data presented in the manuscript are available.
